# Machine Learning Model for Risk Prediction of Community-Acquired Acute Kidney Injury Hospitalization From Electronic Health Records: Development and Validation Study

**DOI:** 10.2196/16903

**Published:** 2020-08-04

**Authors:** Chien-Ning Hsu, Chien-Liang Liu, You-Lin Tain, Chin-Yu Kuo, Yun-Chun Lin

**Affiliations:** 1 Department of Pharmacy Kaohsiung Chang Gung Memorial Hospital Kaohsiung Taiwan; 2 School of Pharmacy Kaohsiung Medical University Kaohsiung Taiwan; 3 Department of Industrial Engineering and Management National Chiao Tung University Hsinchu Taiwan; 4 Division of Pediatric Nephrology Kaohsiung Chang Gung Memorial Hospital and Chang Gung Medical University Kaohsiung Taiwan

**Keywords:** community-acquired acute kidney injury (CA-AKI), hospitalization, treatment decision making, clinical decision support system, machine learning, feature selection with extreme gradient boost (XGBoost), least absolute shrinkage and selection operator (LASSO), risk prediction

## Abstract

**Background:**

Community-acquired acute kidney injury (CA-AKI)-associated hospitalizations impose significant health care needs and contribute to in-hospital mortality. However, most risk prediction models developed to date have focused on AKI in a specific group of patients during hospitalization, and there is limited knowledge on the baseline risk in the general population for preventing CA-AKI-associated hospitalization.

**Objective:**

To gain further insight into risk exploration, the aim of this study was to develop, validate, and establish a scoring system to facilitate health professionals in enabling early recognition and intervention of CA-AKI to prevent permanent kidney damage using different machine-learning techniques.

**Methods:**

A nested case-control study design was employed using electronic health records derived from a group of Chang Gung Memorial Hospitals in Taiwan from 2010 to 2017 to identify 234,867 adults with at least two measures of serum creatinine at hospital admission. Patients were classified into a derivation cohort (2010-2016) and a temporal validation cohort (2017). Patients with the first episode of CA-AKI at hospital admission were classified into the case group and those without CA-AKI were classified in the control group. A total of 47 potential candidate variables, including age, gender, prior use of nephrotoxic medications, Charlson comorbid conditions, commonly measured laboratory results, and recent use of health services, were tested to develop a CA-AKI hospitalization risk model. Permutation-based selection with both the extreme gradient boost (XGBoost) and least absolute shrinkage and selection operator (LASSO) algorithms was performed to determine the top 10 important features for scoring function development.

**Results:**

The discriminative ability of the risk model was assessed by the area under the receiver operating characteristic curve (AUC), and the predictive CA-AKI risk model derived by the logistic regression algorithm achieved an AUC of 0.767 (95% CI 0.764-0.770) on derivation and 0.761 on validation for any stage of AKI, with positive and negative predictive values of 19.2% and 96.1%, respectively. The risk model for prediction of CA-AKI stages 2 and 3 had an AUC value of 0.818 for the validation cohort with positive and negative predictive values of 13.3% and 98.4%, respectively. These metrics were evaluated at a cut-off value of 7.993, which was determined as the threshold to discriminate the risk of AKI.

**Conclusions:**

A machine learning–generated risk score model can identify patients at risk of developing CA-AKI-related hospitalization through a routine care data-driven approach. The validated multivariate risk assessment tool could help clinicians to stratify patients in primary care, and to provide monitoring and early intervention for preventing AKI while improving the quality of AKI care in the general population.

## Introduction

Acute kidney injury (AKI) is defined as an acute increase in serum creatinine (SCr) or reduction in urine volume [1]. Most AKI cases (67%-80%) develop in the community (ie, community-acquired AKI [CA-AKI]), and despite substantial hospitalization care [2-4], CA-AKI is associated with an increased risk of in-hospital mortality compared with that of hospitalized patients without AKI (65%-90%) [2-5]. The financial burden associated with AKI, including the need for dialysis and intensive unit care during hospitalization and the lack of kidney recovery following discharge care, poses significant strain on the health care system [6,7].

The 22nd Acute Disease Quality Initiative Consensus Conference suggested that high-quality care for patients with AKI or those at risk of AKI should start at the community level and continue in the emergency department, hospital setting, and after discharge from inpatient care [8]. AKI is often reversible. The diagnosis of early-stage AKI is difficult as it depends on SCr measurements and urine outputs that are difficult to routinely monitor in outpatient practice. Existing evidence has highlighted the need for clinical tools to provide early and accurate predictions for diagnosing CA-AKI and to deliver preventive management that may prevent irreversible nephron loss in the general population. However, most of the AKI prediction models developed to date focus on a specific group in the hospital setting, such as the risk of hospital-acquired AKI developed following operations [9,10], cardiac procedures [11,12], liver transplantation [13], or intensive care unit admission [14-16]; the few studies assessing risk for the general population are prone to external validity bias.

With significant advances in the application of machine-learning techniques, the extreme gradient boost (XGBoost) [13,14], least absolute shrinkage and selection operator (LASSO) [11,12,15], and random forest [13,16] models have been employed for predicting AKI risk in different clinical scenarios and have shown promising advantages based on the aggregation of data from electronic health records (EHRs). However, current methodological approaches for AKI risk prediction for highly selective groups of patients have limited impact in terms of the rapid integration of such applications into real-world clinical decision support systems. In addition, some AKI prediction models focus on biomarkers that are not widely available for assessment in practice [15]. AKI could be associated with a variety of causes such as nephrotoxins, existing disease status, and volume status. Therefore, a diagnostic tool with routinely measured characteristics and laboratory tests can easily identify patients with a high probability of developing CA-AKI and inform physicians on the possibility of its occurrence. Subsequent attention and action to hemodynamic monitoring and avoidance of nephrotoxins may ultimately enhance care and improve patient outcomes.

The primary aim of this study was to develop and validate a risk prediction model of CA-AKI hospitalization that can be used to identify patients at higher risk of developing CA-AKI and requiring hospital care. Because the XGBoost and LASSO algorithms have been widely and successfully used for predicting the risk of AKI development from EHR data [11-15], both algorithms were employed in this study to further explore machine-learning models for CA-AKI hospitalization risk prediction and gain insights into improving such prediction models. The secondary aim of the study was to transform the prediction model into a scoring function to quantify the risk of CA-AKI hospitalization. This scoring function can facilitate risk assessment and guide treatment decisions for modifiable risk management and prevention in an outpatient setting.

## Methods

### Study Cohort

A nested case-control study was performed on hospitalized patients aged 20 years or older admitted to the emergency department or outpatient clinic and requiring hospitalization between 2010 and 2017 (Multimedia Appendix 1) from a group of Chang Gung Memorial Hospitals (CGMHs) located in different cities from the north to south of Taiwan. The EHR data from CGMHs included 6.1% outpatient and 10.2% inpatient encounters of the Taiwan population in 2015 [17].

Adults hospitalized from 2010 to 2016 served as the training dataset and data from patients hospitalized during 2017 were used in the internal validation model. To estimate the robust probability of CA-AKI associated with hospitalization, patients’ reference (≤3 months) and index (at the admission date) values of SCr were required to determine an acute episode of kidney injury. This study was approved by the Institutional Review and Ethics Board of CGMH, Taoyuan in Taiwan (permit number: 201801461B0). All datasets used in this study were deidentified prior to being transferred to the study investigators. The study followed the Transparent Reporting of a Multivariable Prediction Model for Individual Prognosis or Diagnosis (TRIPOD) statement for reporting multivariable prediction model development and validation [18].

### Predictors

Based on a literature review of factors shown to increase the risk of AKI and expert opinions [19,20], we identified 55 candidate predictor variables (Multimedia Appendix 2), including comorbid conditions, outpatient nephrotoxic medicines, recent emergency department visits, outpatient or hospital admissions, and potential laboratory results during the baseline period within 90 days prior to the index hospital admission (medicines, visits, and laboratory results) and within 365 days preceding the index admission (comorbid conditions).

These diagnosis codes were then classified into 17 binary comorbidity groupings as defined by the Charlson comorbidity index (CCI) [21]. Patients’ concomitant outpatient medicine records were classified into 16 therapeutic binary indicator groupings and counted as the total number of active classes of medicines. Laboratory results included 10 test items in continuous data. These prespecified variables in the initial set of candidate variables for training and validation model prediction algorithms were not limited to strong statistical assumptions of causality or correlation and presumably provided an opportunity to discover new knowledge from machine-learning methods. The full list of candidate variables with corresponding variable names in the data form can be found in Multimedia Appendix 2.

### Outcomes

The outcome was CA-AKI-associated hospitalization defined according to the Kidney Disease: Improving Global Outcomes (KDIGO) criteria as an increase in SCr within 48 hours of 0.3 mg/dl from the reference value, ≥1.5 of the reference value (or increase to 4 mg/dl) within 7 days or up to 90 days prior to the patient’s first admission (index date) in the study period [1]. The reference SCr value was first retrieved based on the availability of measured SCr within 2 days prior to the index date, within 7 days for patients without a recent 2-day SCr, and within 30 days or up to 90 days for patients without any SCr measurements for 7 days. Two approaches were used to determine reference SCr. First, for patients who had multiple SCr measures in the 2- and 7-day time windows, the latest SCr (ie, closest to the index date) was chosen as the reference SCr. Second, the mean SCr value within 8-90 days before the index date was defined as the reference SCr for patients who had multiple SCr measurements in the period [22]. Only patients who had reference and index SCr measures were included for analysis in the study (Multimedia Appendix 1). Patients who fulfilled the KDIGO AKI criteria were classified into the case group and patients without AKI at admission were included in the control group. Stage 1 AKI was defined as an SCr increase of ≥0.3 mg/dl from the reference value within 2 days or an increase of 1.5 to 1.9 of reference SCr within 7 days; stage 2 was defined as an increase of 2.0 to 2.9 of reference SCr; and stage 3 AKI was defined as an increase of ≥3 of reference SCr, an increase to ≥4 mg/dl, or when the patient required dialysis or kidney transplantation for AKI [1].

### Model Development and Validation

**Figure 1:**
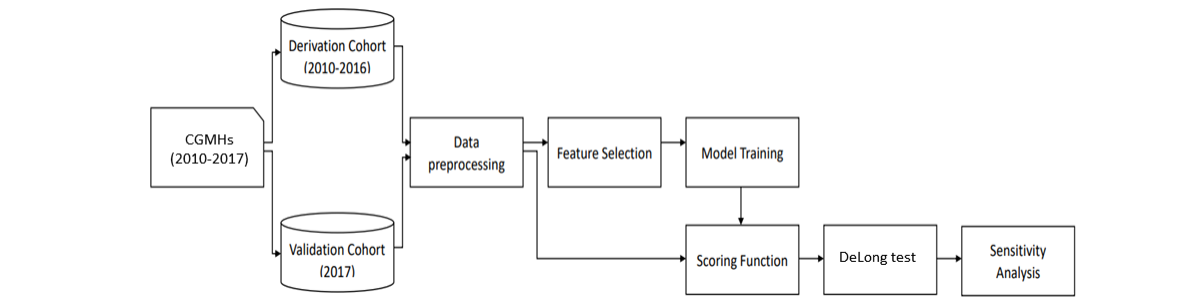
Flowchart for prediction and risk scoring. CGMHs: Chang Gung Memorial Hospitals.

The analytical process included four major stages: preprocessing, feature selection, prediction model construction, and scoring function. The first stage involved the selection of prespecified variables and imputation of missing values. The purpose of the second stage, feature selection, was to select important variables associated with the prediction outcome using state-of-the-art algorithms, XGBoost and LASSO. The third stage involved the construction of a prediction model according to data-driven technology. Finally, we used the model coefficients to build a scoring function, which outputs risk scores based on the prediction results. The whole process is schematically presented in Figure 1.

#### Preprocessing

To estimate the risk of hospitalized patients with CA-AKI at admission for establishing a prevention strategy in an outpatient setting, the model only included predicted variables that were available before hospitalization. Notably, SCr measured on the index hospital admission and the physiological measurements after hospitalization were not included in the model.

Missing values are commonly present in medical records, and dropping medical records or variables with incomplete data would lead to small sample sizes. To develop a more precise model, we selected and discarded the variables with an original missing rate of more than 90% in a step-by-step manner to confirm which variable would significantly contribute to the model even though it had a high missing rate. We considered two approaches to impute the missing continuous values stratified by sex by replacing any missing value with the median or mean of the corresponding group. The experimental results indicated that imputation by the median stratified by sex yields better performance than that of imputation by the mean; thus, we applied imputation by the median stratified by sex to address the problem of missing values. There were no missing data for categorical variables in the dataset. Once the preprocessing step was completed, 47 variables remained for further processing (Table 1).

**Table 1:** Patient characteristics between the derivation and temporal validation cohorts.

Predictor candidates		Derivation cohort (n=204,064)					Temporal validation cohort (n=30,803)			
		n	CA-AKI^a^ (n=17,230)	No CA-AKI (n=186,834)	*P* value^b^	n		CA-AKI (n=2218)	No CA-AKI (n=28,585)	*P* value^b^
Age at index hospitalization (years), mean (SD)			65.26 (15.49)	60.55 (16.23)	<.0001			65.69 (15.98)	60.12 (16.07)	<.0001
**Sex, n (%)**					.003					.21
	Male	93,026	8041 (46.67)	84,985 (45.49)		14,644		1083 (48.83)	13,561 (47.44)	
	Female	111,038	9189 (53.33)	101,849 (54.51)		16,159		1135 (51.17)	15,024 (52.56)	
**Charlson comorbid condition at baseline, n (%)**										
	Acute myocardial infarction	4689	600 (3.48)	4089 (2.19)	<.0001	445		56 (2.52)	389 (1.36)	<.0001
	Congestive heart failure	11,507	1903 (11.04)	9604 (5.14)	<.0001	1368		209 (9.42)	1159 (4.05)	<.0001
	Peripheral vascular diseases	3976	609 (3.53)	3367 (1.80)	<.0001	342		43 (1.94)	299 (1.05)	.0001
	Cerebral vascular accident	22,833	2622 (15.22)	20,211 (10.82)	<.0001	2991		286 (12.89)	2705 (9.46)	<.0001
	Dementia	5101	693 (4.02)	4408 (2.36)	<.0001	382		54 (2.43)	328 (1.15)	<.0001
	Pulmonary disease	18,930	1978 (11.48)	16,952 (9.07)	<.0001	2372		213 (9.60)	2159 (7.55)	<.001
	Rheumatic disease	2387	253 (1.47)	2134 (1.14)	0.0001	437		37 (1.67)	400 (1.40)	.30
	Peptic ulcer	27,709	2966 (17.21)	24,743 (13.24)	<.0001	3597		338 (15.24)	3259 (11.40)	<.0001
	Mild liver diseases	30,682	3217 (18.67)	27,465 (14.70)	<.0001	2382		197 (8.88)	2185 (7.64)	.04
	Diabetes without complication	45,795	6260 (36.33)	39,535 (21.16)	<.0001	5636		696 (31.38)	4940 (17.28)	<.0001
	Diabetes with complications	12,017	2218 (12.87)	9799 (5.24)	<.0001	1863		330 (14.88)	1533 (5.36)	<.0001
	Paraplegia	2484	248 (1.44)	2236 (1.20)	.006	290		19 (0.86)	271 (0.95)	.67
	Renal disease	19,620	5603 (32.52)	14017 (7.50)	<.0001	2867		684 (30.84)	2183 (7.64)	<.0001
	Any malignancy	50,927	4650 (26.99)	46,277 (24.77)	<.0001	6957		603 (27.19)	6354 (22.23)	<.0001
	Severe liver diseases	3439	687 (3.99)	2752 (1.47)	<.0001	209		56 (2.52)	153 (0.54)	<.0001
	Metastatic solid tumor	13,638	1459 (8.47)	12,179 (6.52)	<.0001	1788		199 (8.97)	1589 (5.56)	<.0001
**Prior use of nephrotoxic medicine, n (%)**										
	NSAIDs^c^ or COX II^d^ inhibitors	61,320	4664 (27.07)	56,656 (30.32)	<.0001	8153		531 (23.94)	7622 (26.66)	.005
	Opioid analgesics	14,511	1825 (10.59)	12,686 (6.79)	<.0001	1697		215 (9.69)	1482 (5.18)	<.0001
	Any analgesics	67,782	5587 (32.43)	62,195 (33.29)	.02	8983		642 (28.94)	8341 (29.18)	.81
	Antimicrobials^e^	53,454	5185 (30.09)	48,269 (25.84)	<.0001	7485		641 (28.90)	6844 (23.94)	<.0001
	Antiepileptics (gabapentin or phenytoin)	1441	144 (0.84)	1297 (0.69)	.03	137		12 (0.54)	125 (0.44)	.48
	Renin-angiotensin system inhibitors or potassium-sparing diuretics	50,879	6686 (38.80)	44,193 (23.65)	<.0001	6827		791 (35.66)	6036 (21.12)	<.0001
	Contrast media	14,115	515 (2.99)	13,600 (7.28)	<.0001	2477		76 (3.43)	2401 (8.40)	<.0001
	Nonmetformin OHA^f^	27,476	3780 (21.94)	23,696 (12.68)	<.0001	3678		433 (19.52)	3245 (11.35)	<.0001
	Metformin OHA	14,059	1234 (7.16)	12,825 (6.86)	.14	1774		148 (6.67)	1626 (5.69)	.06
	Any OHA	32,887	4178 (24.25)	28,709 (15.37)	<.0001	4488		505 (22.77)	3983 (13.93)	<.0001
	Immunosuppressants	8100	900 (5.22)	7200 (3.85)	<.0001	1156		137 (6.18)	1019 (3.56)	<.0001
	Antihyperuricemia	9588	1810 (10.50)	7778 (4.16)	<.0001	1232		266 (11.99)	966 (3.38)	<.0001
	Antiinflammation/intestine	1167	75 (0.44)	1092 (0.58)	.01	153		9 (0.41)	144 (0.50)	.53
	Antihistamines, antipsychotics, antispasmodics	37,791	4402 (25.55)	33,389 (17.87)	<.0001	5179		545 (24.57)	4634 (16.21)	<.0001
	Bisphosphonates	822	68 (0.39)	754 (0.40)	.86	107		6 (0.27)	101 (0.35)	.52
	Digoxin	2851	374 (2.17)	2477 (1.33)	<.0001	242		28 (1.26)	214 (0.75)	.008
	Statins	24,818	2660 (15.44)	22,158 (11.86)	<.0001	4158		369 (16.64)	3789 (13.26)	<.0001
	Fibrates	4024	478 (2.77)	3546 (1.90)	<.0001	468		50 (2.25)	418 (1.46)	.003
	Lithium	145	9 (0.05)	136 (0.07)	.33	22		0 (0.00)	22 (0.08)	.19
	Nitrates	12,339	1832 (10.63)	10,507 (5.62)	<.0001	1276		155 (6.99)	1121 (3.92)	<.0001
	Anticoagulants	11,341	1392 (8.08)	9949 (5.33)	<.0001	2244		245 (11.05)	1999 (6.99)	<.0001
**Baseline laboratory result, mean (SD)**										
	SCr^g^	204,064	2.43 (2.61)	1.02 (0.68)	<.0001	30,803		2.15 (2.32)	0.98 (0.59)	<.0001
	eGFR^h^	204,064	66.68 (54.96)	82.47 (34.06)	<.0001	30,803		68.67 (52.78)	83.59 (31.80)	<.0001
	BUN^i^	100,474	36.1 (28.06)	19.06 (14.00)	<.0001	14,674		34.79 (27.56)	18.5 (12.91)	<.0001
	Total cholesterol	17,570	173.6 (39.41)	179.24 (36.74)	<.0001	2499		176.8 (43.40)	177.83 (37.17)	.76
	LDL^j^-cholesterol	57,784	99.46 (31.45)	103.27 (30.43)	<.0001	9452		97.42 (31.78)	102.38 (30.43)	<.001
	Triglyceride	63,600	141.2 (80.91)	134.87 (77.48)	<.0001	9612		140.76 (82.69)	135.1 (78.73)	.05
	Serum uric acid	59,096	7.04 (2.33)	6.3 (1.95)	<.0001	8729		6.52 (2.26)	5.98 (1.86)	<.0001
	Calcium	56182	8.66 (0.76)	8.86 (0.64)	<.0001	7988		8.64 (0.74)	8.92 (0.65)	<.0001
	Phosphorus	36181	4.32 (1.20)	3.59 (0.75)	<.0001	5053		4.28 (1.21)	3.61 (0.73)	<.0001

^a^CA-AKI: community-acquired acute kidney injury.

^b^Independent *t* tests were performed for continuous data, and Pearson Chi-square tests were performed for categorical data in between-groups comparisons.

^c^NSAIDs: nonsteroidal anti-inflammatory drug.

^d^COX II: cyclooxygenase 2.

^e^Antimicrobials include aminoglycosides, penicillins, antivirals, trimoxazole/trimethoprim, fluconazole, teicoplanin/vancomycin, or tetracycline.

^f^OHA: oral hypoglycemic agent.

^g^SCr: serum creatinine.

^h^eGFR: estimated glomerular filtration rate (175 × SCr ^-1.154^ × age^-0.203 12^ × [0.742,female]).

^i^BUN: blood urea nitrogen.

^j^LDL: low-density lipoprotein.

#### Feature Selection

To build a scoring function to help clinicians effectively assess the risk of CA-AKI hospitalization, the number of variables (10) involved in the scoring function was considered based on the commonly used scoring functions in health care and our expert opinion. For instance, there are 14 parameters in the APACHE II score for mortality prediction in a critical care setting (ie, age, temperature, mean atrial pressure, pH, heart rate/pulse, respiratory rate, sodium, potassium, creatinine, acute kidney failure, hematocrit, white blood cell count, Glasgow Coma Scale, and FiO_2_) [23] and 8 parameters in the CHA2DS2VASc score for thromboembolism risk in atrial fibrillation (ie, congestive heart failure, hypertension, age≥75, diabetes, stroke/transient ischemic attack/thrombo-embolism, vascular disease, age 65-74, and sex) [24]. We used the feature selection technique to determine the most important features. An exhaustive search of the best combination of features can conceivably be performed on problems with few features. However, the problem is known to be a nondeterministic polynomial time (NP)-hard problem [25], meaning that the search quickly becomes computationally intractable. Therefore, we used XGBoost and LASSO to perform feature selection.

LASSO is a regression analysis method that uses L1 constraint to perform variable selection and regularization, providing a base to select a subset of the available covariates for use in the final model. XGBoost is an improved algorithm based on the gradient boosting decision tree, which can help avoid model overfitting [26] by considering L1 and L2 constraints in the objective function. The XGBoost model always involves many classification and regression trees, each of which comprises splitting nodes during model learning. Each splitting node corresponds to a variable or feature. This study considered the average gain of the feature when it is used in the trees. The average gain is obtained by calculating the average improvement in accuracy brought about by a feature to the branches it belongs.

#### Prediction Model Construction

After completing feature selection, we used the top 10 important features to build a prediction model for CA-AKI hospitalization. As mentioned above, feature selection is an NP-hard problem. Different methods resulted in the same result for feature selection, demonstrating that the selected features are important from two different perspectives (Multimedia Appendix 3). The performance of prediction models with all, 10, and 5 features was examined to ensure the appropriateness of a 10-features predictive CA-AKI hospitalization risk model (Multimedia Appendix 4).

Next, based on the 10 selected features, we used a logistic regression model as the prediction model because it could reveal the coefficients of the 10 features, which facilitates interpretation for medical personnel to assess the magnitude of the relationship between the individual and outcome variables, and to understand how the outcomes are induced from the model. The ability of model discrimination was determined with the area under the receiver operating characteristic (ROC) curve (AUC). We applied 5-fold crossvalidation to ensure that all data points were used for model training and evaluation so that the obtained model could be generalized to unseen data. Moreover, the 95% CIs for the metrics, including AUC, sensitivity, and specificity, were also determined.

#### Scoring Function

We used the coefficients of the logistic regression model to build a scoring function, which could be used in clinical settings and provide more explanatory power. The outcome of the logistic regression model was the probability of CA-AKI hospitalization. We transformed the probability into a score by multiplying the probability by 100. To distinguish between CA-AKI and non-CA-AKI patients from the scores, it is necessary to determine a threshold value.

We considered two methods to determine the cut-off point for distinguishing between CA-AKI and non-CA-AKI patients. The first method was based on the Youden index [27] to determine the point at which the summation of sensitivity and specificity is maximal, and the second method determined the point that yields the highest sensitivity with a minimum specificity of 0.7 [16]; here, we designate the former as the regular threshold and the latter as the special threshold. With these thresholds, sensitivity, specificity, positive predictive value (PPV), and negative predictive value (NPV) were calculated to analyze the diagnostic ability of the proposed scoring function.

After rescaling the output of the scoring function, we designed an easy-to-use app with an Excel worksheet so that clinicians can estimate the risk score of a patient based on the patient’s relevant data. For missing data entries for a patient, the app uses a default value to perform the estimation, which is obtained from the median of the continuous variable or the mode of the discrete variable stratified by sex.

#### Validation

The temporal validation cohort included hospitalized adults identified during 2017, which was used as the validation set. The scoring function developed in the derivation cohort was applied to the validation cohort to ensure its performance and generalization ability. The AUC and sensitivity values in the validation set were similar to those in the training set, indicating that the proposed method did not suffer from the overfitting problem.

### Statistical Analysis

The DeLong test was applied to assess the generalization ability of the proposed scoring function to ensure the prediction capability to unseen data. We tested the ROC curves of the scoring function on the derivation and validation cohorts, and set the significance level at .05.

### Sensitivity Analysis

Sensitivity analysis was performed to assess the proposed scoring function regarding the discrimination of patients with severe AKI (AKI stage 2-3). In the experimental setting, we relabeled the outcome of the same dataset based on the information on AKI stage. Following the same setting, we divided the dataset into the derivation cohort (2010-2016) and the temporal validation cohort (2017). In the previous setting, the goal was to develop a model to predict whether a patient would suffer from CA-AKI, and therefore separated the whole population into two groups: CA-AKI patients (case) and non-CA-AKI patients (control). In contrast to the previous experiment, the sensitivity analysis included patients without CA-AKI and those with stage 1 CA-AKI in the control group, whereas patients with stage 2 and 3 CA-AKI were included in the case group. We subsequently used the developed scoring function and the same cut-off thresholds to predict the relabeled dataset to investigate whether the scoring function could better distinguish between severe CA-AKI and non-CA-AKI groups.

## Results

### Characteristics of the Study Cohort

A total of 234,867 patients with hospital admission were analyzed in the final CA-AKI cohort; there were 48% admissions from the emergency department and 52% admissions from the outpatient setting between January 1, 2010 and December 31, 2017. The rate of CA-AKI was 8.44% (17,230/204,064) in the derivation cohort and 7.20% (2218/30,803) in the temporal validation cohort (Figure 1). The mean age of patients in the CA-AKI group was higher than that of patients in the non-CA-AKI group in the derivation cohort, which were similar to the temporal validation cohort (Table 1). The frequency of patients with an estimated glomerular filtration rate (eGFR) below 60 ml/min/1.73m^2^ at baseline was higher in the CA-AKI group than that in the non-CA-AKI group (50.67%, 8732/17,230 vs 24.86%, 46,445/186,834) in the derivation cohort, as well as for patients in the validation cohort (46.75%, 1037/2218 vs 21.72%, 6207/28,585). The mean levels of SCr and eGFR at baseline in both the derivation and validation cohorts are presented in Table 1. Compared to the non-CA-AKI group, patients with CA-AKI had a higher mean CCI score (3.08, SD 2.33 vs 1.83, SD 2.02) and had more frequent use of renin-angiotensin system (RAS) inhibitors or diuretics (38.8% vs 23.65%) and antimicrobials (30.09% vs 25.84%) in the baseline period in both the derivation and validation cohorts (Table 1).

### Model Performance

The LASSO and XGBoost models selected the same top 10 important variables among the 47 variables in the derivation cohort, and the AUC (0.789, 95% CI 0.785-0.793) was slightly higher for XGBoost than for the LASSO model (0.7671, 95% CI 0.7621-0.7721) (Multimedia Appendix 3). Table 2 shows the top 10 variables. In the training of the derivation cohort, the logistic regression model had an AUC of 0.7670 (95% CI 0.7608-0.7732), sensitivity of 0.6142 (95% CI 0.5855-0.6431), and specificity of 0.7848 (95% CI 0.7529-0.8167). In addition to the predictive model, the coefficients for the 10 variables used to develop our proposed scoring function are listed in Table 3.

**Table 2:** Top 10 features selected by the extreme gradient boost (XGBoost) and least absolute shrinkage and selection operator (LASSO) algorithms.

Type	Important features
Basic information	Age at index hospitalization
Charlson comorbid condition	Diabetes without complication, Chronic kidney disease, Severe liver diseases
Prior use of nephrotoxic medicine	RAS^a^ inhibitors/K-sparing diuretics
Baseline laboratory result	Serum creatinine, eGFR^b^, BUN^c^, Calcium, Phosphorus

^a^RAS: renin-angiotensin system.

^b^eGFR, estimated glomerular filtration rate (175 × SCr − 1.154 × age − 0.203 × [0.742, female]).

^c^BUN: blood urea nitrogen.

**Table 3:** Community-acquired acute kidney injury risk coefficients in the final model.^a^

Variable (Xi)	Coefficient (βi)
SCr^b^	0.7244
Age	0.0207
eGFR^c^	0.0169
BUN^d^	0.0072
Calcium	–0.4669
Phosphorus	0.3542
DM^e^	0.3065
CKD^f^	0.6235
SLD^g^	0.9647
RAS^h^ inhibitors/ K-sparing diuretics	0.4099

^a^Intercept of the model: –3.6838.

^b^SCr: serum creatinine.

^c^eGFR: estimated glomerular filtration rate.

^d^BUN: blood urea nitrogen.

^e^DM: diabetes without complication.

^f^CKD: chronic kidney disease.

^g^SLD: severe liver disease.

^h^RAS: renin-angiotensin system.

### Scoring Function for CA-AKI Hospitalization

The scoring function was established based on the coefficients obtained from the logistic regression model. Equation (1) shows the formula of the scoring function *z*, in which βi is the coefficient for the *i*th feature *Xi*. Detailed definitions of the variables and their corresponding coefficients are presented in Table 3. The final score was obtained by transforming the value of *z* into a probability with a sigmoid function, and then multiplying it by 100 to make the risk score range from 0 to 100.


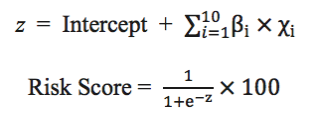


**Figure 2:**
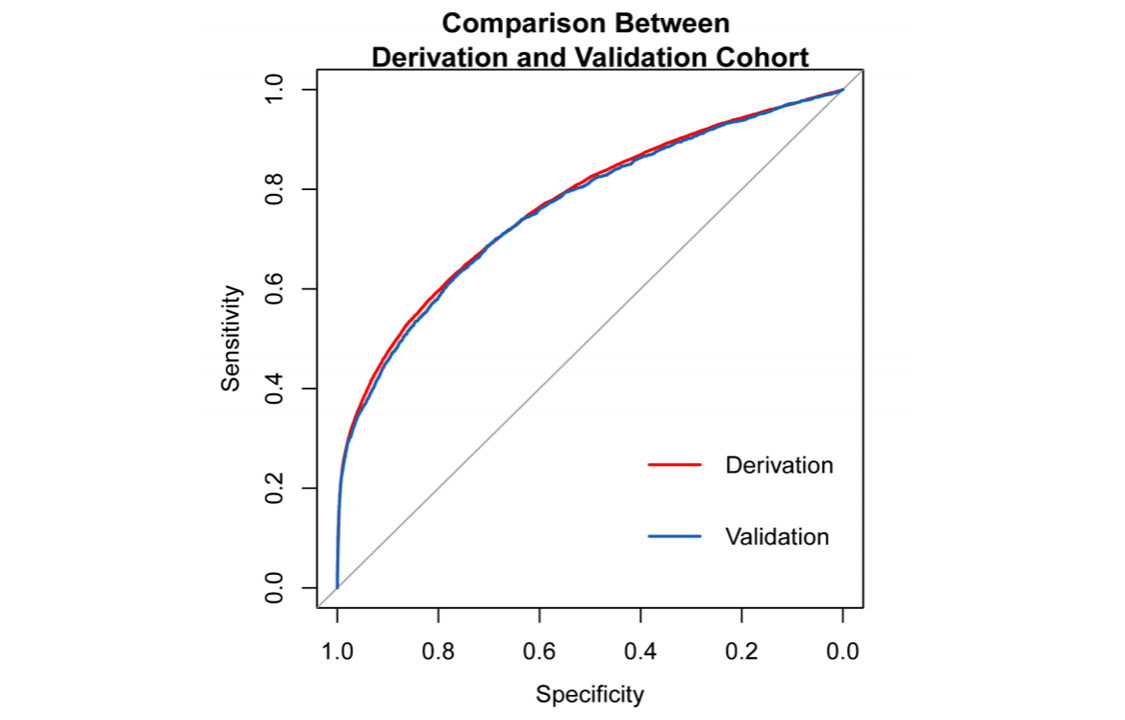
DeLong test for the receiver operating characteristic curves of derivation and validation cohorts.

**Figure 3:**
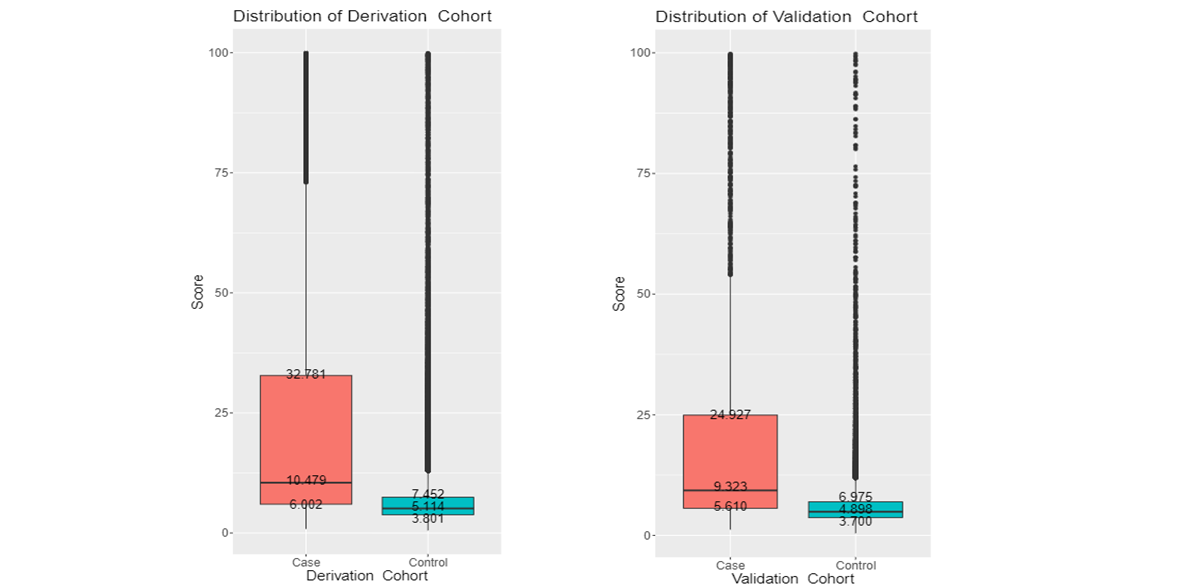
Risk score distribution. Left: Derivation cohort with CA-AKI stages 1-3 (case). Right: Validation cohort with CA-AKI stages 1-3 (case).

To verify the generalization ability of the proposed scoring function, the DeLong test was applied to the ROC curves of the derivation and validation cohorts. As shown in Figure 2, the *P* value of the test was approximately .30, indicating no statistically significant difference between the ROC curves of the derivation and validation cohorts and that the scoring function does not suffer from the overfitting problem. A higher score indicates a higher the risk of CA-AKI hospitalization. Figure 3 shows the risk score distributions between the case (CA-AKI) and control (non-CA-AKI) groups for the derivation and validation cohorts. The experimental results indicated that the risk score of the case group is normally higher than that of the control group in the derivation and validation cohorts, meaning that the scoring function could stratify the two groups well.

**Figure 4:**
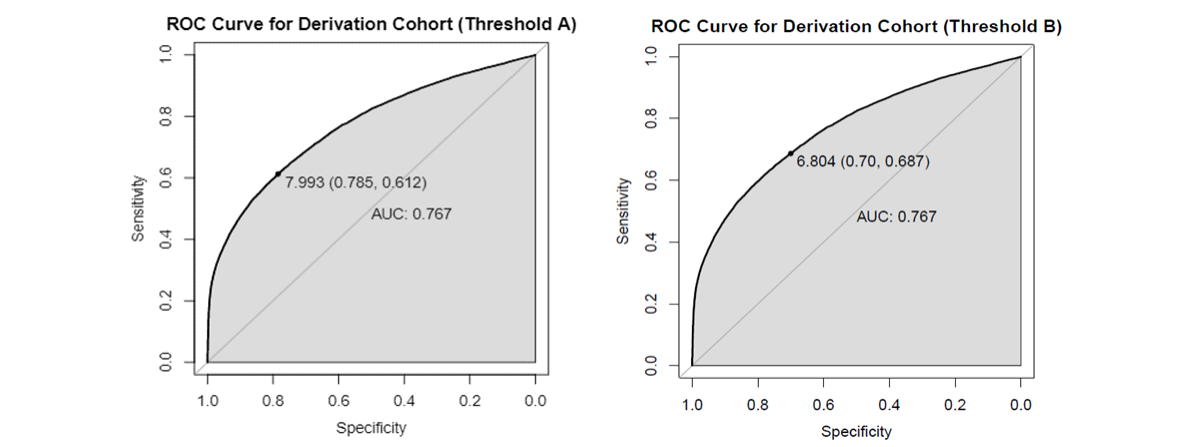
Receiver operating characteristic (ROC) curve for the derivation cohort. Threshold A: Cut-off regular threshold value of 7.993; Threshold B: Cut-off special threshold value of 6.804.

Table 4 shows the results of model performance, demonstrating that the scoring function could achieve better results in sensitivity and NPV by setting a special threshold as the cut-off point. The ROC curves for the cut-off thresholds determined by the two methods are presented in Figure 4, in which the values in parentheses are specificity and sensitivity. Finally, the risk equation for CA-AKI hospitalization risk was established in an Excel worksheet (Multimedia Appendix 5) to allow for automatic computation by importing patient information in the clinical decision support system.

**Table 4:** Model performance in the derivation and validation cohorts.

Performance metric^a^	Cut-off point with regular threshold (7.993)			Cut-off point with special threshold (6.804)			
	CA-AKI^b^ stages 1-3		CA-AKI stages 2 and 3		CA-AKI stages 1-3		CA-AKI stages 2 and 3
	Derivation cohort	Validation cohort	Validation cohort		Derivation cohort	Validation cohort	Validation cohort
AUC^c^	0.767 (0.758-0.777)^d^	0.761	0.818		0.767 (0.758-0.777)	0.761	0.818
Sensitivity	0.612 (0.591-0.634)	0.569	0.689		0.687 (0.665-0.708)	0.651	0.75
Specificity	0.785 (0.782-0.788)	0.814	0.807		0.700 (0.694-0.706)	0.736	0.728
PPV^e^	0.208 (0.201-0.215)	0.192	0.133		0.174 (0.169-0.180)	0.161	0.106
NPV^f^	0.956 (0.954-0.959)	0.961	0.984		0.960 (0.958-0.963)	0.964	0.985

^a^The performance for each cohort was evaluated based on disease severity and cut-off threshold values.

^b^CA-AKI: community-acquired acute kidney injury.

^c^AUC: area under the receiver operating characteristic curve.

^d^The values in the parentheses are 95% CIs calculated through 5-fold crossvalidation.

^e^PPV: positive predictive value.

^f^NPV: negative predictive value.

### Sensitivity Analysis

**Figure 5:**
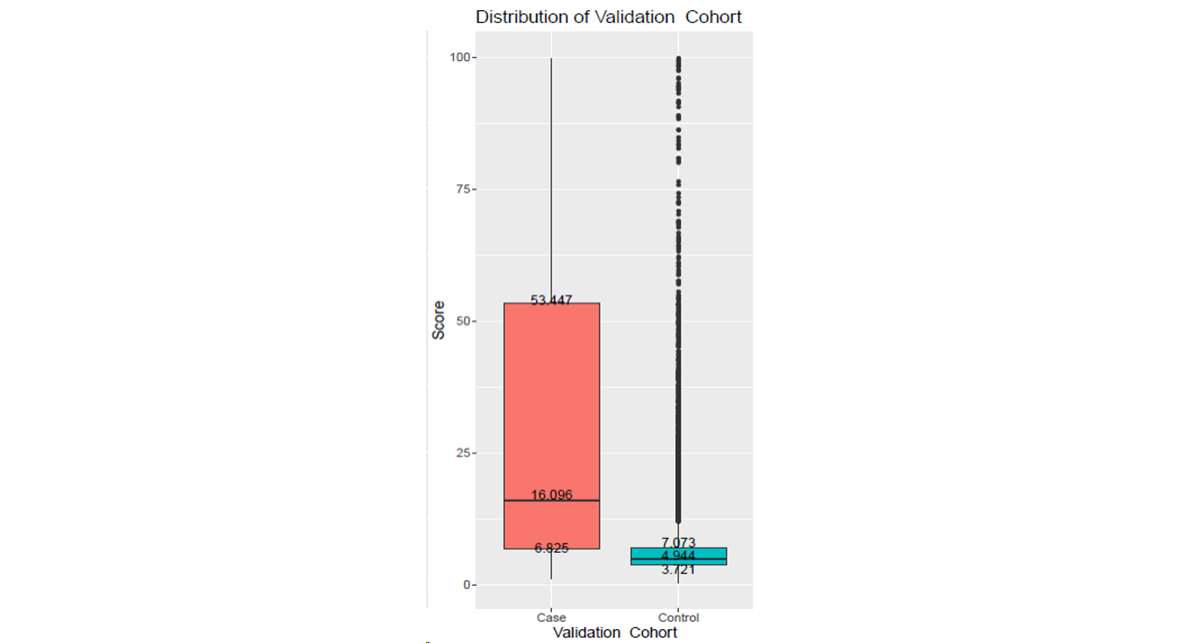
Risk score distribution of the validation cohort with stage 2-3 community-acquired-acute kidney injury (case).

The sensitivity analysis was conducted with the original model using the relabeled validation data to evaluate model performance in distinguishing between severe AKI and less severe AKI cases. The risk score distribution between the case and control groups is depicted in Figure 5, which is more distinguishable. Moreover, sensitivity and specificity were more balanced compared to the original values, regardless of using the regular threshold (7.993) or special threshold (6.804) as the cut-off value in Figure 4. The experimental results are shown in Table 4. Using the cut-off special threshold, the AUC value was 0.818, sensitivity was 0.75, and NPV was 0.985 in the temporal validation cohort, which were all better than the original values, except for PPV, indicating that the proposed scoring function performed better in distinguishing severe CA-AKI.

## Discussion

### Principal Findings

To the best of our knowledge, this is the first study to use a machine-learning model to develop a 10-variable scoring function for assessing the risk of CA-AKI hospitalization. The advantages of the predictive risk model include prediction based on routinely available EHR data in practice and full applicability to general patients in an outpatient setting. Most importantly, the quantified risk score can serve as an assessment tool to support preventive management for the risks of CA-AKI hospitalization that can be modified. The two proposed methods, XGBoost and LASSO, selected identical top 10 features from different perspectives to support the importance of these predictors in the scoring function.

LASSO is a statistical method that can improve prediction accuracy and model interpretation by imposing an L1 penalty, resulting in a sparse model. Notably, LASSO shrinks some model coefficients to zero, providing a base to eliminate variables whose coefficients are not statistically different from zero. In contrast, XGBoost is an ensemble machine-learning model involving multiple decision trees, and it can estimate feature importance by considering the contribution of a specific feature for each tree during the learning process.

Importantly, XGBoost reflects the diverse etiologies of CA-AKI (eg, physiological status of the patient, coexisting medical problems, and underlying causes) affecting the general population. The experimental results indicated that using the proposed scoring function for assessing the risk of CA-AKI hospitalization showed fairly good to good performance with respect to the AUC (0.76-0.82) to detect any stage or moderate to severe stages of CA-AKI by decision thresholds on the validation models.

Similar to previous AKI investigations, underlying comorbidities (diabetes mellitus, severe liver diseases, chronic kidney disease) [28,29] and recent use of RAS inhibitors or potassium-sparing diuretics [19] were identified in the present CA-AKI hospitalization risk score. The risk model also indicated that low calcium levels and high phosphorus levels were potentially modifiable predictors and could be targeted for correction [30]. This feature has substantial clinical implications because it demonstrates that the model can be applied to prospectively support clinical decision systems in real time for rapid screening and recognition of patients with a predicted risk of CA-AKI in outpatient settings.

Early-stage AKI is generally asymptomatic, and therefore SCr monitoring is required. Previous findings have suggested that even small changes are common and are associated with increased mortality and length of hospital stay [31]. However, because a baseline SCr measurement is not always available in practice settings, risk assessment for mild CA-AKI hospitalization in a diverse population can be a challenge. The present study is one of the few CA-AKI studies that included patients with an SCr measurement in the community as the baseline level of renal function, which was compared to another SCr measurement performed at hospital admission or requested by the general practitioner to better define the nature of CA-AKI [3,4,32,33]. In this study, patients with CA-AKI in the final cohort were older (65.26, SD 15.49 years) than those in the non-CA-AKI group; moreover, 54.4% of the patients were women and 50.67% had preexisting chronic kidney disease (eGFR<60 ml/min/1.73m^2^ at baseline). In comparison, a study with a British population reported a mean age of 74.4 (SD 15.4) years, 50%-52% female patients, and 31.9%-34.6% of patients with preexisting chronic kidney disease [4,30], whereas another study with a US population reported a mean age of 67.8 (SD 12.2) years and 43.2% of patients with preexisting chronic kidney disease [3]; these populations were considered to be comparable with the CA-AKI patients identified using KDIGO SCr-based criteria in different populations. The present study used large-scale clinical data with a representative and adequate sample size to develop a diagnostic tool for CA-AKI risk evaluation.

Because of a lack of data regarding the risk modeling of CA-AKI that included up to a few hundred cases, we summarized the major findings in large, TRIPOD-adherent studies (published after 2010) that have analyzed the risk of AKI development in the short term (within 3 or 7 days) following general admission (see Multimedia Appendix 6) [34-36]. In those studies, variable candidates were first selected according to significance (usually *P*<.05) in the univariate analysis and determined in the multivariate logistic regression analysis with stepwise selection. The handling of missing data in the variables was usually not addressed in these previous risk models. The AUC value was calculated in both training and validation data to indicate the capability of discrimination. Hosmer Lemeshow analysis and *P* values were nonsignificant, suggesting acceptable calibration.

A prediction model for the risk of AKI 72 hours following admission was established using data from 3 centers in the United Kingdom [34]. Based on 35 variables collected within 24 hours after admission in 2011, 12 predictors were selected, including age, primary diagnosis, previous admissions, CCI score, HbA1C, troponin, proteinuria, eGFR, potassium, magnesium, C-reactive protein, and white blood cell count. The AUC in the derivation cohort (n=6626) was 0.67 (95% CI 0.64-0.71) in the internal validation cohort for any AKI risk and was 0.71 (95% CI 0.67-0.76) in the external validation model (n=1585) [34].

Another prospective study using prospectively collected AKI screening data in a single center in the United Kingdom in 2011 predicted the risk of AKI less than 7 days after admission [35]. The baseline SCr was retrieved between 1 and 6 months prior to hospitalization. Of the 25 collected variables, the following 7 variables were selected in the multivariate logistic model: age (60-79, ≥80 years), congestive cardiac failure, chronic kidney disease, diabetes, liver disease, respiratory rate ≥20/min, alert, verbal, pain, unresponsive status. The AUC value was 0.72 (95% CI 0.66-0.77) and 0.76 (95% CI 0.71-0.82) for the derivation and internal validation cohort, respectively. A recent external validation study in a single UK nonspecialist acute hospital (2013-2015) reported that the AUC of the prediction model was 0.65 (95%CI 0.62-0.67) in the medical setting and was 0.66 (95%CI 0.62-0.70) in the surgical setting [36]. In addition, the sensitivity analysis showed that for patients without baseline SCr information across the medical and surgical cohorts, the AUC was 0.71 (95%CI 0.67-0.75) and 0.68 (0.58-0.75), respectively, indicating poor to fair predictive capability (range 0.65-0.71) [36].

### Strengths and Limitations

The current risk prediction model included patients with CA-AKI at hospital admission with modifiable and nonmodifiable predictors commonly measured in routine care. Excluding SCr on the index hospital admission, XGBoost and LASSO demonstrated the feasibility of using machine learning for predicting CA-AKI hospitalization risk in the general population. The present model, which used large-scale clinical data with a representative and adequate sample size and top 10 important predictors having clinical significance on prevention of CA-AKI requiring inpatient care, can be considered as a benchmark for further evaluations.

Another strength of the present risk model is that the continuous risk score of the model can be incorporated into clinical decision support systems, facilitating their usability. Because the CA-AKI hospitalization rate was considerably low in the present study cohort, a risk score over 7.993 was associated with a low PPV (20%) but high NPV (96%), suggesting an ability to correctly identify low-risk patients (ruled out). For instance, the CA-AKI hospitalization risk equation can be easily fitted with the most recent real-time clinical data for automatic screening and implementation of preventive strategies (ie, to stop nephrotoxic medication or ordering nephrologists referred care) in general outpatient and emergency department settings. Recently, a digital-based AKI care pathway incorporating mobile phone detection with a multidisciplinary care response team and care protocol was proposed for the UK National Health System, which showed significant practical value in the field [37]. Furthermore, the results of implementing the CA-AKI risk score in practical settings can help to prospectively evaluate its impact on the quality of patient care, such as reducing AKI risk exposure, preventive measures, and management to avoid renal insults.

This study has several limitations. First, it is based on data obtained from a large hospital cohort in Taiwan. This could affect the generalizability of the study findings, although based on previous data, patient characteristics included in the current study are not different from those of other populations. Similar to previous retrospective studies, medical histories relied on previously coded events in EHRs, and thus possible residual risk may have been underestimated in the baseline period, thereby increasing the uncertainty of the risk prediction performance. Biomarkers of kidney injury such as cystatin C and neutrophil gelatinase-associated lipocalin, which are important to provide diagnostic information but are not routinely tested in practice settings, were absent from the developed model in the present study. Additionally, the study did not address external validation. Although it is clear that the integration of different EHR systems remains a challenge to the medical community in many health systems, including Taiwan, it is still necessary to conduct further studies to leverage the risk scoring system to available EHRs. External validations using this prediction model outside of a study setting and in different geographic populations are envisioned as future work. Lastly, the current study focused on the feasibility of using a machine-learning model for CA-AKI hospitalization risk prediction, but did not examine the incidence of CA-AKI, cause, and management of CA-AKI or its implementation on patient care. The prediction model and risk scoring function developed in the present study can nevertheless serve as a risk assessment tool and link clinical decision support systems for prospective validation.

### Conclusion

This study demonstrated that the selected variables were truly crucial for predicting the risk of CA-AKI hospitalization, as both XGBoost and LASSO identified the same top 10 important variables. The discrimination ability of the CA-AKI risk scoring function was good with an AUC value of 0.76 and 0.82 in the detection of CA-AKI hospitalization at any stage and for moderate to severe stages, respectively, according to decision thresholds on the validation cohort, and suggested the feasibility of AKI detection and prevention in wider populations in the community. In addition, the easy-to-use risk calculator can facilitate its widespread implementation in daily routines and workflows for patient-centered care and prospective validation of machine-learning applications.

## Appendix

Multimedia Appendix 1 Patient selection process.

Multimedia Appendix 2 Data variables used in the machine learning models.

Multimedia Appendix 3 Number of predictors and AUC of XGBoost models.

Multimedia Appendix 4 The top 10 important variables and model performance between XGBoost and LASSO algorithms.

Multimedia Appendix 5 CA-AKI hospitalization risk calculator.

Multimedia Appendix 6 Traditional prediction models for AKI developed in an acute medical setting or shortly after general admission.

## Abbreviations

AKI: acute kidney injury
AUC: area under the receiver operating characteristic curve
CA-AKI: Community-acquired acute kidney injury
CCI: Charlson comorbidity index
CGMHs: Chang Gung Memorial Hospitals
eGFR: estimated glomerular filtration rate
EHR: electronic health records
KDIGO: Kidney Disease: Improving Global Outcomes
LASSO: least absolute shrinkage and selection operator
NP: nondeterministic polynomial time
NPV: negative predictive value
PPV: positive predictive value
RAS: renin-angiotensin system
ROC: receiver operating characteristic
SCr: serum creatinine
TRIPOD: Transparent Reporting of a Multivariable Prediction Model for Individual Prognosis or Diagnosis
XGBoost: extreme gradient boost
