# Supplementary material for: Machine Learning Model for Risk Prediction of Community-Acquired Acute Kidney Injury Hospitalization From Electronic Health Records: Development and Validation Study
Source: J Med Internet Res. 2020 Aug 4;22(8):e16903. doi: 10.2196/16903 (PMC7435690; doi:10.2196/16903)
Supplement: Multimedia Appendix 1 [file jmir_v22i8e16903_app1.pdf]

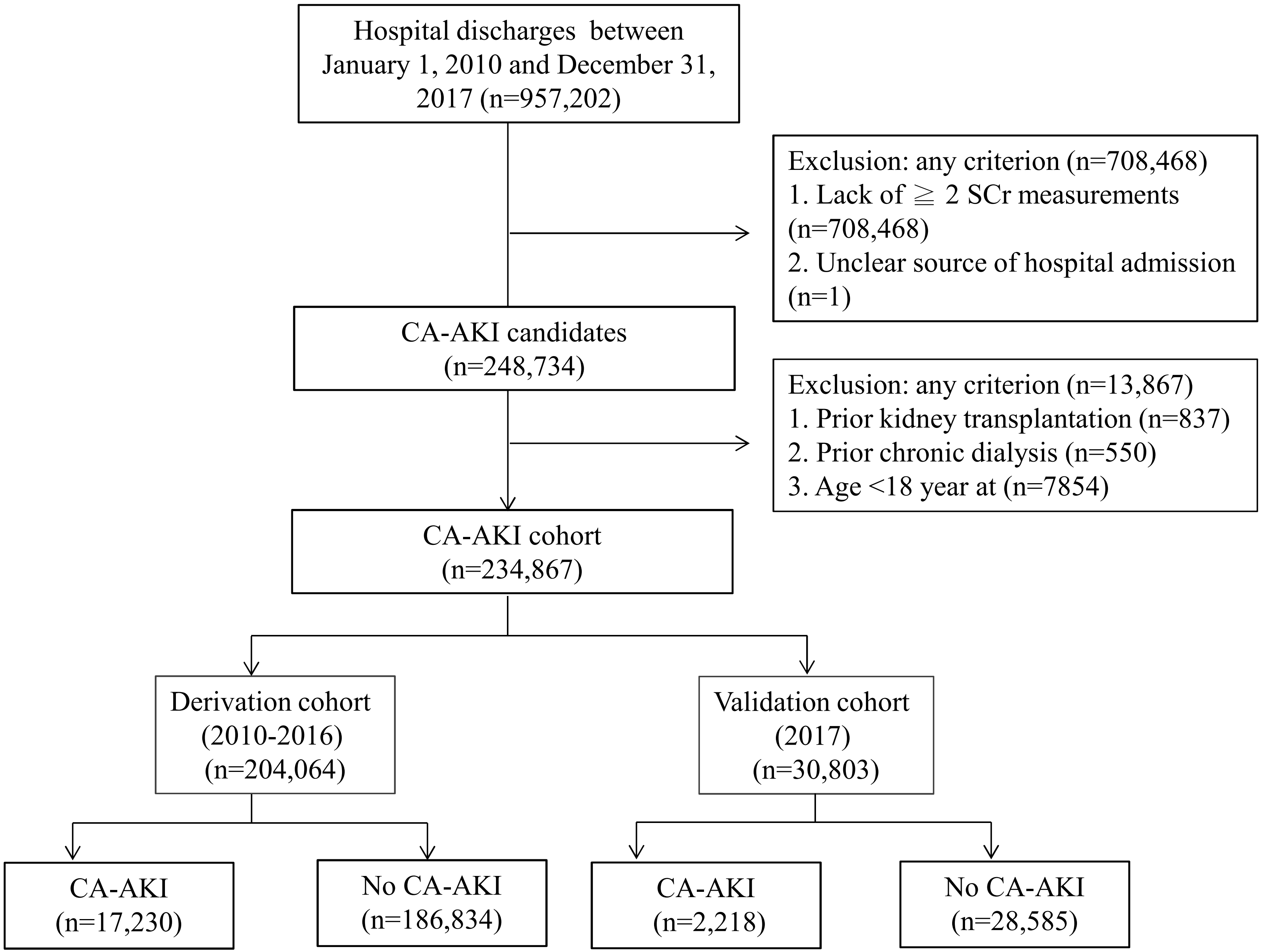


Multimedia Appendix 1. Patient selection process.

SCr= serum creatinine; CA-AKI= community-acquired acute kidney injury
