# Supplementary material for: Machine Learning Model for Risk Prediction of Community-Acquired Acute Kidney Injury Hospitalization From Electronic Health Records: Development and Validation Study
Source: J Med Internet Res. 2020 Aug 4;22(8):e16903. doi: 10.2196/16903 (PMC7435690; doi:10.2196/16903)
Supplement: Multimedia Appendix 2 [file jmir_v22i8e16903_app2.docx]

Multimedia Appendix 2. Data variables used in the machine learning models

| Dimension (number of variable) | Variables (level of measurement) |
| --- | --- |
| Demographics (2) | 1.Age at index hospitalization (continuous data), 2.Gender (binary data, female versus male) |
| Charlson Comorbidities Index diseases (17) | 1.Acute myocardial infarction, 2.Congestive Heart Failure, 3.Peripheral vascular disease, 4.Cerebral vascular accident, 5.Dementia, 6.Pulmonary disease, 7.Connective tissue disorder, 8.Peptic ulcer, 9.Liver disease, 10.Diabetes, 11.Diabetes with complications, 12.Paraplegia, 13.Renal disease, 14.Cancer, 15.Metastatic Cancer, 16.Severe liver disease, 17.HIV or AIDS (all binary data) |
| Therapeutic Medication Classes (21)  19 classes of medications | 1.Nonsteroidal anti-inflammatory drugs or cyclooxygenase enzymes II inhibitors, 2.Opioid analgesics, 3.Antimicrobials (Aminoglycosides or Penicillins or Anti-virals or Co-trimoxazole /Trimethoprim or Fluconazole or Teicoplanin/Vacomycin or Tetracycline), 4.Anti-epileptics (Gabapentin or Phenytoin), 5.Anti-hypertensive agents (Renin–angiotensin system inhibitors or Diuretics – potassium sparing), 6.Contrast media, 7.Non-metformin oral anti-hyperglycemics, 8.Metoformin oral anti-hyperglycemics, 9. [Calcineurin inhibitors](https://www.whocc.no/atc_ddd_index/?code=L04AD&showdescription=no) /Methotrexate, 10.Anti-hyperuricemia, 11.Intestinal Anti-inflammation, 12.Antihistamines or Antipsychotics or Antispasmodics, 13.Bisphosphonates, 14.Digoxin, 15.Statins, 16.Fibrates, 17.Lithium, 18.Nitrates, 19.Anti-coagulants (binary data: use versus no use ), 20. sum of Antimicrobials (continuous), 21 sum of class (continuous) |
| Laboratory results (11) | 1.Serum creatinine, 2.estimated glomerular filtration rate*, 3.BUN, 4.Serum uric acid, 5.Cholesterol-LDL, 6.Total-cholesterol, 7.HbA1C, 8.Triglyceride, 9.Potassium, 10.Calcium, 11.Phosphorus, (all continuous data) |
| Health service utilization (4) | 1.Number of outpatient visit (continuous), 2.Number of emergency department visit (continuous), 3.Number of hospitalization (continuous), 4.Prior dialysis (binary data: yes versus no) within 3 months prior to the index hospitalization |

A variable with missing data ≥90% was excluded from the pre-processing procedure (only 47 variables were included in feature selection, Table 1).

*eGFR, estimated glomerular filtration rate was calculated by the equation=(175 ×SCr ^-1.154^ × age^-0.203 12^ × [0.742,female])

**References:**

# Sundararajan V, Henderson T, [Perry C](https://www.ncbi.nlm.nih.gov/pubmed/?term=Perry%20C%5BAuthor%5D&cauthor=true&cauthor_uid=15617955), Muggivan A, [Quan H](https://www.ncbi.nlm.nih.gov/pubmed/?term=Quan%20H%5BAuthor%5D&cauthor=true&cauthor_uid=15617955), [Ghali WA](https://www.ncbi.nlm.nih.gov/pubmed/?term=Ghali%20WA%5BAuthor%5D&cauthor=true&cauthor_uid=15617955). New ICD-10 version of the Charlson comorbidity index predicted in-hospital mortality. [*J Clin Epidemiol.*](https://www.ncbi.nlm.nih.gov/pubmed/15617955) 2004;57(12):1288-94.

Dreischulte T, Morales DR, Bell S, Guthrie B. Combined use of nonsteroidal anti-inflammatory drugs with diuretics and/or renin–angiotensin system inhibitors in the community increases the risk of acute kidney injury. Kidney international. 2015;88(2):396-403.

Murugan R, Weissfeld L, Yende S, Singbartl K, Angus DC, Kellum JA. Association of statin use with risk and outcome of acute kidney injury in community-acquired pneumonia. Clinical journal of the American Society of Nephrology : CJASN. 2012;7(6):8[1]95-905.

ROSNER & OKUSA. Drug-associated acute kidney injury in the intensive care unit. In: de Broe, M.E., Portere, G.A. editors. Clinical nephrotoxins : Renal Injury from Drugs and Chemicals. Springer; 2008, XVII, 987p., Hardcover.
