# Supplementary material for: Machine Learning Model for Risk Prediction of Community-Acquired Acute Kidney Injury Hospitalization From Electronic Health Records: Development and Validation Study
Source: J Med Internet Res. 2020 Aug 4;22(8):e16903. doi: 10.2196/16903 (PMC7435690; doi:10.2196/16903)
Supplement: Multimedia Appendix 3 [file jmir_v22i8e16903_app3.docx]

Multimedia Appendix 3. The top 10 important variables and model performance between XGBoost and LASSO algorithms

| Predictive Model : XGBoost | |  | Predictive Model : LASSO | |
| --- | --- | --- | --- | --- |
| AUC | 0.789±0.004 |  | AUC | 0.7671±0.005 |
| Sensitivity | 0.651±0.017 |  | Sensitivity | 0.5977±0.022 |
| Specificity | 0.7764±0.0205 |  | Specificity | 0.8026±0.022 |
| **names** | **importance** |  | **names** | **importance** |
| Baseline SCr | 100 |  | Baseline SCr | 100 |
| Baseline eGFR | 18.18 |  | Baseline eGFR | 75.54 |
| Baseline BUN | 11.12 |  | Age at admission | 34.88 |
| Baseline Calcium | 8.47 |  | CKD | 13.9 |
| CKD | 5.18 |  | RASI/ Diuretics | 12.51 |
| Age at admission | 4.08 |  | Baseline Calcium | 10.44 |
| RASI/Diuretics | 3.62 |  | Baseline Phosphorus | 7.11 |
| Baseline Phosphorus | 3.38 |  | Diabetes without complications | 5.4 |
| Diabetes without complications | 0.62 |  | Severe liver disease | 4.93 |
| Severe liver disease | 0 |  | Baseline BUN | 0 |

RASI=Renin-angiotensin system inhibitors/aldosterone inhibitor diuretics
