# Supplementary material for: Machine Learning Model for Risk Prediction of Community-Acquired Acute Kidney Injury Hospitalization From Electronic Health Records: Development and Validation Study
Source: J Med Internet Res. 2020 Aug 4;22(8):e16903. doi: 10.2196/16903 (PMC7435690; doi:10.2196/16903)
Supplement: Multimedia Appendix 4 [file jmir_v22i8e16903_app4.docx]

Multimedia Appendix 4. Number of predictors and AUC of XGBoost models

| XGBoost model  with all (47) variables | |  | XGBoost model  with top 10 variables | |  | XGBoost model with top 5 variables | |
| --- | --- | --- | --- | --- | --- | --- | --- |
| AUC | 0.8011±0.0093 |  | AUC | 0.789±0.004 |  | AUC | 0.7438±0.0045 |
| Sensitivity | 0.6706±0.064 |  | Sensitivity | 0.651±0.017 |  | Sensitivity | 0.542±0.0235 |
| Specificity | 0.7715±0.0661 |  | Specificity | 0.7764±0.0205 |  | Specificity | 0.8321±0.0278 |
| **names** | **importance** |  | **names** | **importance** |  | **names** | **importance** |
| Baseline SCr | 100 |  | Baseline SCr | 100 |  | Baseline SCr | 100 |
| Baseline eGFR | 59.67 |  | Baseline eGFR | 18.18 |  | Baseline eGFR | 57.98 |
| Baseline BUN | 16.02 |  | Baseline BUN | 11.12 |  | Age at admission | 30.57 |
| Baseline Calcium | 12.41 |  | Baseline Calcium | 8.47 |  | Baseline Calcium | 6.48 |
| Age at admission | 9.46 |  | CKD | 5.18 |  | Baseline BUN | 0 |
| CKD | 8.55 |  | Age at admission | 4.08 |  |  |  |
| RASI/Diuretics | 6.77 |  | RASI/Diuretics | 3.62 |  |  |  |
| Baseline Phosphorus | 5.97 |  | Baseline Phosphorus | 3.38 |  |  |  |
| Diabetes without complications | 3.53 |  | Diabetes without complications | 0.62 |  |  |  |
| Severe liver disease | 3.18 |  | Severe liver disease | 0 |  |  |  |
| Baseline serum uric acid | 2.59 |  |  |  |  |  |  |
| Any tumor | 2.37 |  |  |  |  |  |  |
| Baseline HbA1c | 2.26 |  |  |  |  |  |  |
| Baseline triglycerides | 1.65 |  |  |  |  |  |  |
| Anti-cholinergic agents | 1.57 |  |  |  |  |  |  |
| Sum of class of anti-microbial agents (1-7) | 1.5 |  |  |  |  |  |  |
| Contrast media | 1.47 |  |  |  |  |  |  |
| Metastatic solid tumor | 1.18 |  |  |  |  |  |  |
| Opioid analgesics | 1.14 |  |  |  |  |  |  |
| Baseline Cholesterol-LDL | 1.12 |  |  |  |  |  |  |
| Calcineurin inhibitors/Methotrexate | 1.08 |  |  |  |  |  |  |
| Mild liver disease | 0.84 |  |  |  |  |  |  |
| Anti-gout agents | 0.63 |  |  |  |  |  |  |
| Nonsteroidal antiinflammatory drugs | 0.54 |  |  |  |  |  |  |
| Diabetes with complications | 0.5 |  |  |  |  |  |  |
| Congestive heart failure | 0.36 |  |  |  |  |  |  |
| Anticoagulants | 0.29 |  |  |  |  |  |  |
| Dementia | 0.2 |  |  |  |  |  |  |
| Prior dialysis (not chronic dialysis) | 0.2 |  |  |  |  |  |  |
| Ulcer disease | 0.2 |  |  |  |  |  |  |
| Peripheral vascular disease | 0.2 |  |  |  |  |  |  |
| Myocardial infarction | 0.19 |  |  |  |  |  |  |
| Nitrates | 0.17 |  |  |  |  |  |  |
| Chronic obstructive disease | 0.16 |  |  |  |  |  |  |
| Male (vs female) | 0.14 |  |  |  |  |  |  |
| Cerebrovascular disease | 0.13 |  |  |  |  |  |  |
| Non-metformin anti-diabetic agents | 0.13 |  |  |  |  |  |  |
| Rheumatic disease | 0.11 |  |  |  |  |  |  |
| Metformin anti-diabetic agents | 0.1 |  |  |  |  |  |  |
| Digoxin | 0.08 |  |  |  |  |  |  |
| Hemiplegia | 0.06 |  |  |  |  |  |  |
| Statins | 0.04 |  |  |  |  |  |  |
| Fibrates | 0.03 |  |  |  |  |  |  |
| Anti-epileptics | 0.01 |  |  |  |  |  |  |
| Anti-inflammation_intestine | 0 |  |  |  |  |  |  |
| Bisphosphonates | 0 |  |  |  |  |  |  |
| Lithium | 0 |  |  |  |  |  |  |
