# Supplementary material for: Machine Learning Model for Risk Prediction of Community-Acquired Acute Kidney Injury Hospitalization From Electronic Health Records: Development and Validation Study
Source: J Med Internet Res. 2020 Aug 4;22(8):e16903. doi: 10.2196/16903 (PMC7435690; doi:10.2196/16903)
Supplement: Multimedia Appendix 5 [file jmir_v22i8e16903_app5.pdf]

Risk Calculators for CA-AKI hospitalization

Enter patient's information:

SEX	○ Male<br>◉ Female		Estimated risk of AKI	2.02

*necessary

*Please press *delete* if there is no related data.

		Unit			Unit
SCr		mg/dL	CKD	0	
eGFR		mL/min/1.73m2	Age	47	year
BUN	14.8	mg/dL	RAS inhibitors/ Diuretics*	0	
Calcium	9.4	mg/dL	Diabetes without complication		
Phosphorus	3.3	mg/dL	*Diuretics- Potassium sparing		

Multimedia Appendix 5. CA-AKI hospitalization risk calculator on Excel worksheet.
