# Supplementary material for: Machine Learning Model for Risk Prediction of Community-Acquired Acute Kidney Injury Hospitalization From Electronic Health Records: Development and Validation Study
Source: J Med Internet Res. 2020 Aug 4;22(8):e16903. doi: 10.2196/16903 (PMC7435690; doi:10.2196/16903)
Supplement: Multimedia Appendix 6 [file jmir_v22i8e16903_app6.docx]

Multimedia Appendix 6. Traditional prediction models for AKI developed at acute medical setting or shortly after the general admission

| **Sources** | **Prediction outcome** | **Study population/ setting** | **Model performance** | **Candidate predictors** | **Justification** |
| --- | --- | --- | --- | --- | --- |
| Bedford M et al. (2016) | AKI on admission  Secondary outcomes: AKI stage 2/3 | Derivation cohort (n=27,532 admissions);   - 2 of 3 selected centers in UK (2011) | - 12 variables in the final model: age, primary diagnosis, previous admissions, Charlson score, HbA1C, troponin, proteinuria, eGFR, potassium, magnesium, C-reactive protein and white blood cell - AUC for any AKI   0.75 (0.74-0.77) ; HL test, P= 0.009.   - AUC for AKI 2/3 stage 0.75 (0.73-0.78); HL test, P= 0.0003. | - 35 variables on univariate analysis, entered into backward selection of multivariate logistic regression analysis if P <0.05. - Hosmer-Lemeshow (HL) test, p<0.05 indicated poor fit of a model. - Variables were collected within 24 hours after admission. | - Missing data excluded or given own category; - Model performed better for the any AKI prediction than for the prediction of AKI stage 2/3 |
|  |  | Internal validation cohort  (n=9,157 admissions); same settings in the derivation cohort | - AUROC 0.72 (0.71-0.74) for any AKI; HL test: P=0.12; - AUROC 0.71 (0.68-0.75) for AKI 2/3 stage; HP test, P=0.005 |  |  |
|  |  | External validation cohort (n= 4,726 admissions)   - 1 of 3 selected centers in UK | - AUROC 0.71 (0.67-0.76) for AKI; HL test, P=0.12; - AUROC 0.63 (0.52-0.75) for AKI 2/3 stages; HL test, P=0.14 |  |  |
| Forni LG, et al. (2013);  Hodgson LE et al. (2017) | AKI< 7 days post medical admission (Pre-admission SCr >1 month & <6 months prior) | Derivation cohort (n=1867)   - Single center, UK - Exclusions: RRT, non-medical setting, age <18, AKI on admission (n=184), missing data (n=553). | - 7 Variables: age 60-79, ≥80, CCF, CKD, Diabetes, liver disease, respiratory rate ≥20/min, alert on AVPU. - AUROC 0.72 (0.66–0.77); HL test, P=0.96 | - 25 variables on univariate analysis, entered into multivariable logistic analysis if P <0.05. - Variables were collected at admission: age, respiratory rate, consciousness scale AVPU (alert, vocal, pain, unresponsive,); history of chronic kidney disease (CKD), diabetes, congestive cardiac failure (CCF) and liver disease (n=7) | - Excluding AKI on admission; No baseline SCr values, only normal SCr at admission; variables with missing data; - Providing predictive risk score |
|  |  | Internal validation cohort (n=1656), same setting in derivation cohort. | AUROC 0.76 (0.71–0.82) |  |  |
|  |  | External validation cohort* (2013–2015, n=12554)   - Single UK non-specialist acute hospital; - Exclusions: AKI on admission, aged<18; obstetrics/gynaecology admissions; discharged without spending a night in hospital. | - AUROC (Medical setting, patients with baseline SCr): 0.65 (0.62-0.67); HL test, P=0.064 - AUROC (Medical): 0.71 (0.67-0.75); HL test, p=0.014. - AUROC (Surgical setting, patients with baseline SCr): 0.66 (0.62-0.69); HL test, p=0.093; - AUROC (Surgical): 0.67 (0.58-0.75); HL test, p=0.664 | Four settings: medical, general surgical, patients with and without preadmission SCr | With and without baseline SCr; Providing predictive risk score;  sensitivity analysis |

| **References:**  Bedford M, Stevens P, Coulton S, et al. Development of risk models for the prediction of new or worsening acute kidney injury on or during hospital admission: a cohort and nested study. Southampton, UK: Queen’s Printer and Controller of HMSO. 2016.  Forni LG, Dawes T, Sinclair H, Cheek E, Bewick V, Dennis M, et al. Identifying the patient at risk of acute kidney injury: a predictive scoring system for the development of acute kidney injury in acute medical patients. Nephron Clinical practice. 2013;123(3-4):143-50.  Hodgson LE, Dimitrov BD, Roderick PJ, Venn R, Forni LG. Predicting AKI in emergency admissions: an external validation study of the acute kidney injury prediction score (APS). BMJ open. 2017;7(3):e013511. |
| --- |

Hosmer-Lemeshow tests p values (P>0.05) were non-significant, suggesting acceptable calibration for the model.
